# Tests of evolutionary and genetic rescue using flour beetles, *Tribolium castaneum*, experimentally evolved to thermal conditions

**DOI:** 10.1002/ece3.11313

**Published:** 2024-04-29

**Authors:** Rebecca Lewis, Michael D. Pointer, Lucy Friend, Matthew J. G. Gage, Lewis G. Spurgin

**Affiliations:** ^1^ School of Biological Sciences University of East Anglia Norwich UK

**Keywords:** genetic rescue, inbreeding, small populations, *Tribolium*

## Abstract

Small, isolated populations are often characterised by low levels of genetic diversity. This can result in inbreeding depression and reduced capacity to adapt to changes in the environment, and therefore higher risk of extinction. However, sometimes these populations can be rescued if allowed to increase in size or if migrants enter, bringing in new allelic variation and thus increasing genetic diversity. This study uses experimental manipulation of population size and migration to quantify their effects on fitness in a challenging environment to better understand genetic rescue. Using small, replicated populations of *Tribolium castaneum* experimentally evolved to different temperature regimes we tested genetic and demographic rescue, by performing large‐scale manipulations of population size and migration and examining fitness consequences over multiple generations. We measured fitness in high temperature (38°C) thermal lines maintained at their usual ‘small’ population size of *N* = 100 individuals, and with ‘large’ scaled up duplicates containing *N*≈10,000 individuals. We compared these large lines with and without migration (*m* = 0.1) for 10 generations. Additionally, we assessed the effects of outcrossing at an individual level, by comparing fitness of hybrid (thermal line × stock) offspring with within‐line crosses. We found that, at the population level, a rapid increase in the number of individuals in the population resulted in reduced fitness (represented by reproductive output and survival through heatwave conditions), regardless of migration. However, at an individual level, the hybrid offspring of migrants with native individuals generally demonstrated increased longevity in high temperature conditions compared with individuals from thermal selection lines. Overall, these populations showed no evidence that demographic manipulations led to genetic or evolutionary rescue. Following the effects of migration in individuals over several generations may be the next step in unravelling these conflicting results. We discuss these findings in the context of conservation intervention.

## Graphical abstract

We use experimental evolution to test the effects of demographic and evolutionary rescue in populations of *Tribolium castaneum* previously adapted to high temperature and kept at small population sizes. We find no evidence of rescue in these populations and discuss the results in the context of conservation intervention.

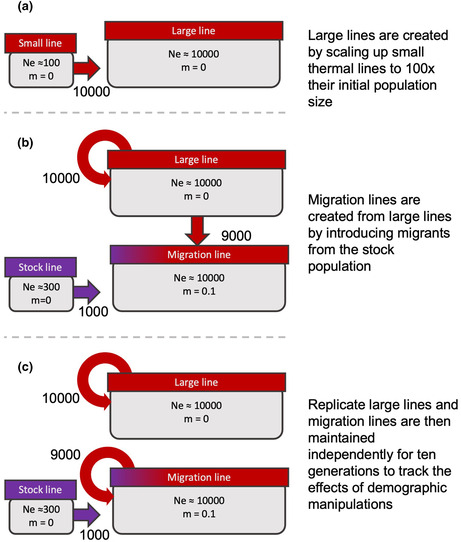

## 1 INTRODUCTION

Human activity has had dramatic impacts on the environment, including higher global average temperatures and habitat destruction, which can lead to species extinction (Pimm, 2008). The outcome of these pressures is that populations are increasingly confined to small patchy habitats, resulting in population fragmentation and smaller, more isolated populations (Gibbs, 2001). In such a changing world, small populations are at risk of extinction. Smaller populations generally have lower genetic diversity due to increased rates of genetic drift (Frankham, 1996), resulting in a reduced adaptive potential (Hoffmann et al., 2017). Another feature of small populations is that, for any given individual, the pool of potential mates is smaller than that of a large population, leading to increased levels of inbreeding and inbreeding depression (Wright, 1977). Thereby, small populations can have reduced levels of genetic diversity, with negative consequences for individual fitness and population persistence, particularly in changing environments (Durkee et al., 2024; Lande, 1988) and in the context of widespread anthropogenic disturbance (Ceballos et al., 2017; Lande, 1999; Young et al., 2016).

While in many cases, populations will go extinct once they drop to a certain size (e.g. due to the extinction vortex; Gilpin, 1986), there are ways in which populations can be ‘rescued’. Evolutionary rescue is the restoration of a population through adaptation to novel conditions following environmental change (Bell, 2013; Carlson et al., 2014; Gomulkiewicz & Holt, 1995). Evidence in support of evolutionary rescue comes both from the wild and from laboratory studies (Bell & Gonzalez, 2009; Gomulkiewicz & Shaw, 2013; Hufbauer et al., 2015). Another route to genetic restoration is through the introduction of migrant individuals from conspecific populations, which is expected, through outcrossing, to increase genetic diversity, individual fitness, and population growth rates (Ingvarsson, 2001). This process is known as genetic rescue, and is again supported by experimental and observational studies (e.g. Åkesson et al., 2016; Bouzat et al., 2009; Schwartz & Mills, 2005). One well‐known example of genetic rescue occurred following a natural migration event in which a single male wolf (*Canis lupus*) joined a struggling population in Scandinavia (Vilà et al., 2003), with resulting offspring exhibiting increased fitness and decreased inbreeding coefficients (Åkesson et al., 2016). Such natural migrations, alongside examples of conservation translocations (e.g. Johnson et al., 2010), show the potential for assisted migration to be used as a conservation tool for endangered populations, to boost genetic diversity, population size and fitness. Indeed, assisted migration has been used successfully as a conservation tool in a number of taxa (e.g. Florida Panthers, *Puma concolor coryi*, Mansfield & Land, 2002; bighorn sheep, *Ovis canadensis*, Hogg et al., 2006; and European adders, *Vipera berus*, Madsen et al., 1999).

Despite the popularity of assisted migration as a conservation tool to bring about genetic rescue, it has been argued that migration into small populations can also have negative effects (Tallmon et al., 2004). The introduction of migrants can result in outbreeding depression, defined as a reduction in fitness as a result of outcrossing (Lynch, 1991). The most commonly cited cause of outbreeding depression is that outcrossing can break down local adaptation, which can occur when there is high genetic and/or environmental divergence between outcrossing populations (Banes et al., 2016; Lenormand, 2002; Montalvo & Ellstrand, 2001). It has also been suggested that migration of outbred individuals into a small population introduces genetic load, recessive deleterious variants whose effects are masked in the outbred population, but become expressed in the small population when they become homozygous under inbreeding, resulting in lower average fitness (Lynch & O'Hely, 2001). For this reason, conservation managers seek a high level of confidence that any demographic manipulations would not be to the detriment of endangered populations. The outcomes of rescue are dependent upon the interaction between the genetic makeup of source and sink populations, therefore, a detailed understanding of the demographic and adaptive processes that underlie genome evolution is required in order to ensure that conservation measures result in successful genetic restoration of populations (Hedrick, 2005).

Experimental evolution can provide a platform to evaluate how genetic rescue operates in a controlled manner and to understand its impacts over different time scales (Frankham, 2015; Tallmon et al., 2004). Because natural genetic rescue events are either, by their nature, one‐off observations or conservation events in endangered populations, laboratory experiments on model species provide the only empirical approach for testing genetic rescue parameters in a replicated manner, without putting endangered populations at risk. *Tribolium castaneum*, the model organism for this study, is well suited for transgenerational genetic rescue research. *T. castaneum* populations can be reared in large numbers and have short generation times, so can be monitored over several generations following a genetic rescue event, on a much shorter timescale than is possible in most natural populations. Additionally, *T. castaneum* has long been a model for population dynamics (King & Dawson, 1972; Pointer et al., 2020), including how genetic processes affect population demographics (e.g. Desharnais & Liu, 1987; Durkee et al., 2024; Hufbauer et al., 2015; Pray et al., 2009; Wade & Goodnight, 1991). Previous work in this system has shown the vulnerability to the extinction of small inbred populations (Durkee et al., 2024) and demonstrated the potential for evolutionary, genetic and demographic rescue in populations newly introduced to challenging environments (Durkee et al., 2024; Hufbauer et al., 2015). However, the effects of demographic changes on small populations pre‐exposed to challenging conditions are not understood.

In this study, we experimentally investigate the impact of demographic manipulations on the fitness of previously small and inbred *T. castaneum* populations which have been evolving at 38°C for 52 generations. In previous work, no increase in the fitness of lines exposed to 38°C has been observed (Lewis et al., in preparation), which may be due to the small population sizes at which they are maintained (*n* = 100), allowing the negative effects of inbreeding depression to swamp the positive effects of thermal adaptation. We test whether demographic manipulations of population size and migration rate are able to increase fitness in these populations. By combining migration from an outbred population with an increase in population size we provide opportunity for pre‐existing thermally adaptive variation to recombine with new variation introduced by migrants. By increasing population sizes, we aim to increase the number of recombination events per generation, and thereby the number of haplotypes generated, which could then be acted upon by selection by keeping populations at a thermally challenging temperature. To test this at a population level, we experimentally manipulated the population size and migration rates, and compared how these manipulations affected fitness. One suggested way that genetic rescue might be more likely to occur is via long‐term genetic restoration (Hedrick, 2005), rather than a single rescue event (Adams et al., 2011), therefore, we maintained these demographic manipulations over 10 generations to allow time for the effects of introgression and recombination to be observable. To complement these experiments, and to study the effects of outbreeding at the individual level, we quantified the fitness of crosses between individuals from small thermally selected populations with individuals from different genetic backgrounds. A common criticism of genetic rescue is that migrants may not be adapted to the environment of the recipient population, which could cause a loss of local adaptation (Storfer, 1999). By also assessing the fitness of crosses between replicate thermal lines (genetically isolated for >50 generations), we could model outcrossing independently of the confounding effect of maladapted migrants. Thus, if an increased recombination of the existing variation alone is able to increase fitness we expect to see large lines performing better in fitness assays than small thermal lines at 38°C. If introgression of new variants is required we expect to see the fitness of migration lines and crosses between small thermal lines being higher than that of either small thermal lines or large lines. If migration of environmentally naive migrants is maladaptive, we expect no increase or possibly a reduction in fitness in migration lines, but may still see a positive effect of crosses between small thermal lines. The results of these assays are discussed in the context of genetic rescue and species conservation.

## 2 METHODS

### 2.1 *Tribolium* stocks, husbandry and the assay procedure

Beetles used were of an outbred stock population known as the Krakow Super Strain (KSS), created in 2008 to combine global *T. castaneum* diversity (Dickinson, 2018; Michalczyk, 2008). The KSS stock populations are maintained at a population size of 600 individuals. The standard husbandry cycle used throughout this study comprised a mating and oviposition phase, followed by a development phase. On day 0 of the cycle, adults selected to found the following generation were removed from their population and placed onto fresh fodder for 7 days of mating and oviposition, after which they were sieved from the fodder and discarded. This then began the 35 day development phase, during which time individuals matured from eggs to adulthood. This regime of non‐overlapping generations is commonly used in *Tribolium* studies (e.g. Hufbauer et al., 2015) as it prevents intergenerational interactions, allows accurate tracking of passing generations and more closely models breeding systems observed in organisms relevant to conservation genetics. The standard fodder medium consisted of 10% brewer's yeast and 90% organic, strong white bread flour, topped with a thin layer of oats to aid traction on the surface. Standard environmental conditions, used for stocks and control populations, were 30°C and 60% relative humidity (RH), with a 12:12 h light: dark cycle (light from 08:00 to 20:00), maintained within the Controlled Environment Facility at the University of East Anglia.

To obtain virgin beetles, individuals were sexed as pupae 12 days after the end of the oviposition phase (at control temperature), and raised in single sex, single line groups of 20. When experiments required setting up of single‐pair matings, mature males in their single‐sex groups were marked with a coloured dot of paint on the dorsal thorax to allow subsequent identification. Male–female pairs at 12 ± 2 days post‐eclosion were then placed in 7 mL plastic vials containing 1.5 g of standard fodder for 24 hours of mating opportunity, following which males were discarded and females transferred to 7 g of fresh fodder in Petri dishes (4.5 cm diameter, 1.5 cm depth) to oviposit. In some experiments females were transferred to a second Petri containing fresh fodder for a second block of oviposition. Reproductive fitness was measured by counting the adult offspring from each mating that had developed in these Petri dishes after a development period.

### 2.2 Thermal selection lines

The thermal selection lines were established from the KSS stock in 2010 (Dickinson, 2018). These lines are maintained at control (30°C) and high (38°C) temperatures at 60% RH, for >52 generations, with 14 independently replicated populations per treatment. The optimal temperature for population productivity under laboratory conditions in *T. castaneum* is 32°C (Howe, 1962), measured by egg hatch rate and larval development rate. Therefore, the control temperature is 2°C below the optimal temperature, and the high temperature regime is 6°C above the optimal temperature. This control temperature was chosen in order to minimise selection pressures in control lines, as this is the temperature at which the stocks used to found thermal lines have been maintained over many generations. This is also a common temperature used for maintaining *T. castaneum* stocks, offering the best opportunity to compare results across lab groups. The high temperature of 38°C was chosen to impose a strong selection pressure and reduce reproductive fitness (Sales, 2018; Sales et al., 2018), while also being below the thermal fertility limit for *T. castaneum*. Every generation, exactly 100 adults go into the mating and oviposition phase, both this and the development phase take place at the line's assigned temperature. The development phase is shorter in high‐ than control temperature lines, due to *T. castaneum* having a reduced development time at 38°C, likely due to an increase in metabolic rate (Calderwood, 1961). As a result, the number of generations of selection differs between the regimes, being 52 generations for 30°C lines and 57 generations for 38°C lines. However, we do not anticipate this discrepancy in generation number to affect the results of any experiments, as 30°C is a control treatment, not expected to impose any stress or selective pressure.

### 2.3 Population‐level demographic manipulations and fitness

**FIGURE 1:**
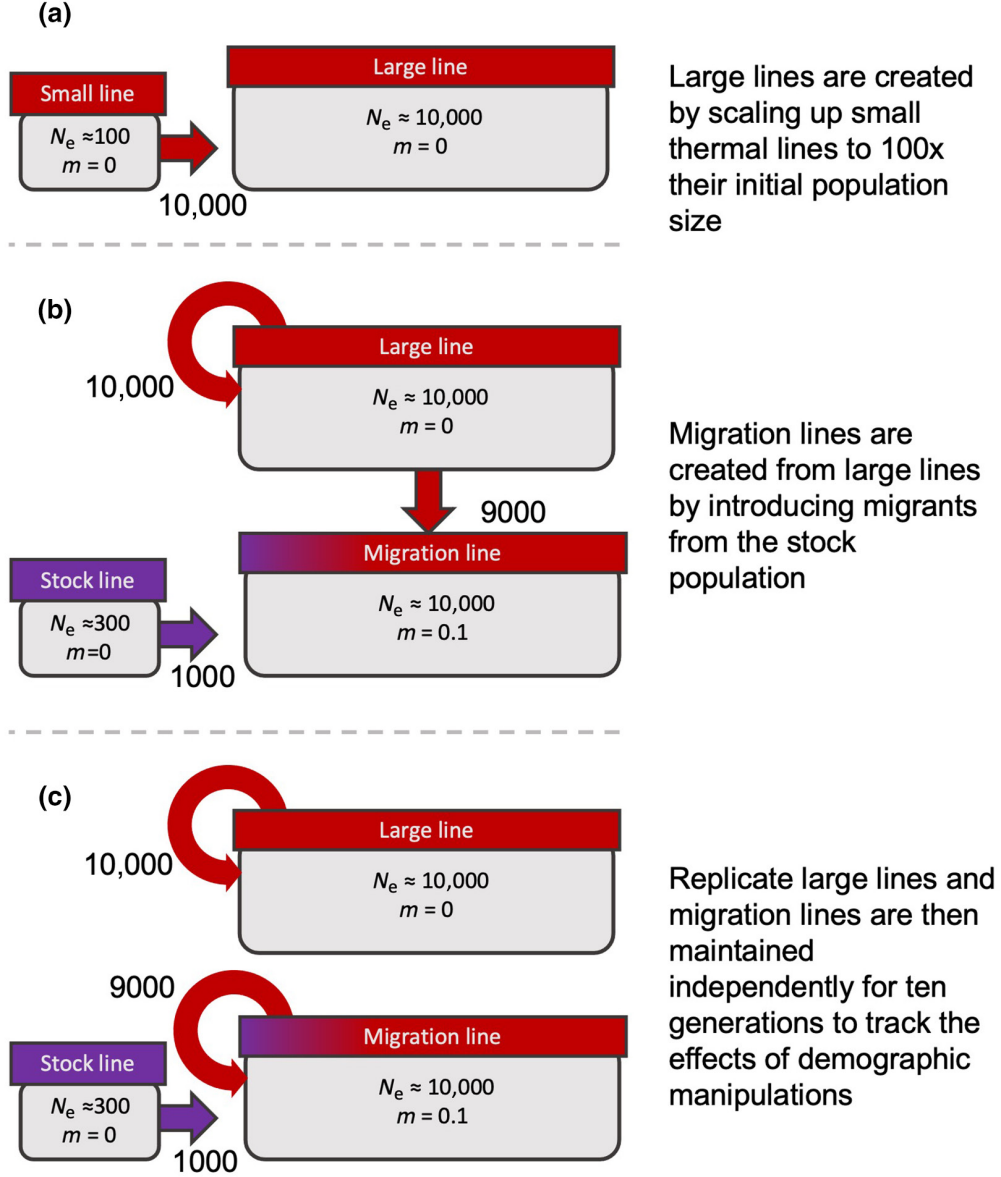
Procedure for creation and maintenance of demographic treatments, showing how (a) large lines were created by scaling up small lines (previously adapted to 38°C) to *N*_e_≈10,000, (b) migration lines were created with *N*_e_≈10,000 by combining 9000 individuals from large lines with 1000 ‘migrants’ from an unselected stock population, (c) large and migration lines were maintained over 10 generations until fitness assays were performed.

To simulate genetic rescue at the population level we created sets of ‘large’ and ‘migration’ thermal lines, within which either recombination or a combination of recombination and introgression could generate new genetic combinations. We then maintained these demographic treatments for 10 generations at the thermally challenging 38°C to allow selection to act on recombined haplotypes, before assaying fitness in these lines. To create the demographic treatment lines, at generation 52, we randomly selected three replicate populations from the original 38°C thermal selection lines (population size of 100, henceforth referred to as ‘small lines’) and used 100 individuals to found a new line, which was scaled up to a population size of 10,000 over two or three generations, depending on the number of offspring produced each generation (Figure 1a). Note that this was done only for 38°C lines – as this is a demographic not a thermal manipulation the controls for this experiment are the small 38°C lines. Once the desired population size was reached, ~10,000 adults were selected each generation (by volume, ~50 mL of adult beetles) to enter the mating and oviposition phase of the husbandry cycle and produce the following generation. These ‘large lines’ were kept in plastic containers measuring approximately 60 × 75 × 21 cm, and containing ~7 kg of fodder to maintain approximately equal population densities to the original, small lines. At generation 55, the large lines were divided and one of each resultant pair was used to create ‘migration lines’, in which gene flow was introduced at a rate of *m* = 0.1 each generation (Figure 1b). To this end, 9000 adults from generation n of the large lines were combined with 1000 adults from the KSS stock population to sire generation *n* + 1. Line maintenance procedures were otherwise identical to the standard cycle described above and all large and migration lines were maintained at 38°C.

To test whether demographic treatments had an effect on population fitness at 30°C and 38°C, we conducted both reproductive output and heatwave survival assays on each population of small, large and migration lines after 10 generations of demographic treatment (Figure 1c). Following the mating and oviposition phase of the husbandry cycle, the egg‐containing fodder was split in two, one half was put into 30°C to develop and the other into 38°C – these different rearing temperatures were used to represent the ancestral environment and the environment to which thermal lines had been exposed for 52 generations. After 16 days (at 30°C) or 12 days (at 38°C) pupae were selected, single‐pair matings set up and reproductive assays conducted over two 7‐day blocks of oviposition as described above, all at the assigned treatment temperature. The sample sizes were ~30 pairs per treatment.

We also carried out heatwave survival assays at generation 65. At reproductive maturity, approximately 300 adults from each replicate line (measured by volume, ~1.5 mL beetles) were placed into a 200 mL container (7 cm diameter × 7.5 cm depth) with a 100 g standard fodder and placed into an incubator at 42°C and 60% RH. These heatwave conditions are at the limits of stock *T. castaneum* survival. Each day, the beetles were sieved out of the tubs, dead beetles were counted and disposed of and live beetles returned to the tubs and placed back into the incubator at 42°C. When five or fewer beetles remained alive in a population, they were transferred to smaller (4.5 cm diameter, 1.5 cm depth) petri dishes containing a 7 g fodder for the remainder of the experiment. The experiment continued until no beetles remained alive. This assay therefore measured survival rates through time under heatwave conditions.

### 2.4 Outcrossing and fitness

To measure the benefits of outcrossing at an individual level and in the short term, we carried out crosses using individuals from the small, long‐term thermal lines and the outbred KSS stocks, and measured the reproductive output of the crosses and the longevity of the offspring. A total of 20 male and 20 female pupae were collected from each thermal line (10 replicate lines at 38°C and 10 at 30°C) and 100 each of male and female pupae were collected from the outbred KSS stock. These were split into single‐sex groups of 20 and allowed to eclose either at their native temperature or the opposite temperature. Ten days post‐eclosion, at reproductive maturity, male and female beetles were paired according to three treatments: (i) thermal line male × thermal line female; (ii) KSS female × thermal male and (iii) thermal female × KSS male. Each treatment was replicated six times per line (control: 30°C, or high: 38°C), giving a total of 360 crosses, 301 of which produced offspring (155 with 30°C lines and 146 with 38°C lines). Reproductive fitness of pairs was assessed using the method outlined in Section 2.1, with oviposition across a single 10‐day block, and development time of 28 days at 38°C or 35 days at 30°C.

In conjunction with counting these offspring, the first 20 beetles counted were put into a petri dish to undergo a longevity assay at 38°C, to measure adult survival under high temperature conditions. This was repeated for the next 20 beetles. The third set of 20 beetles counted were put into a petri dish at 40°C. The outcome of this was that for each male × female pair described above, the longevity of their offspring was tested at 38°C (*n* = 40) and at 40°C (*n* = 20), contingent upon their producing at least 60 offsprings. Each petri dish was filled with 20 beetles before the next was used and any remaining offspring were counted and discarded. At regular intervals the number of deaths per petri dish for that time period were recorded, and the surviving beetles were put into fresh fodder and returned to the assay. The measurement intervals were adjusted between seven and 14 days during the course of the assay in response to observed death rate.

Finally, we assessed the fitness of experimental crosses between individuals from within and between the small, high temperature lines (as in the hybrid experiment, above). This allowed us to investigate the effect of outcrossing independent of the potentially confounding effect of introducing maladaptive variation from a population non adapted to the temperature regime. Ten replicate lines were used in this experiment. The lines were paired and these five pairs were labelled as groups A–E. Crosses between lines were within these five groups. According to the method outlined in Section 2.1, single pair matings were set up wherein females from each line were paired with males from their own line (*n* = 4) or from the other line in the group (*n* = 4). Reproductive fitness was assessed as described in Section 2.1 for each pair across a 7‐day block of oviposition, with offspring having 35 days to develop before freezing and counting.

### 2.5 Statistical analyses

Data analyses were carried out using R version 3.3.1 (R core team, 2016), implemented in RStudio version 1.0.136 (2009–2016). All generalised linear mixed models (GLMMs) were conducted using the ‘lme4’ package (Bates et al., 2015).

To test for differences in reproductive output between demographic treatments, a GLMM was fitted. The number of offspring produced by individual pairs was fitted as the response variable using a Poisson error distribution, and explanatory variables were the demographic treatment (categorical – small/large/migration) and the rearing temperature (categorical –30°C vs. 38°C). The ‘large’ demographic treatment was fitted as the baseline in order to separately identify the effects of population size and migration. The line ID of the mating pair was fitted as a random effect, and an observation level random effect was included to account for overdispersion (Harrison, 2014). To model the interaction between the explanatory variables, a separate model was constructed, as above, but with an interaction between rearing temperature and demographic treatment and from these models only the interaction terms were reported.

All survival analyses were conducted using the ‘survival’ (Therneau, 2015) and ‘coxme’ (Therneau, 2018) packages. To test for differences in survival through a 42°C heatwave between the different demographic treatments, a Cox mixed effects model was fitted which contained rearing temperature and demographic treatment as fixed predictors, alongside random effects of experimental line. To model the interaction between the explanatory variables, a separate model was constructed, as above, but with an interaction factor between rearing temperature and demographic treatment. This experiment was continued until all individuals had died, so there was no requirement for censoring. A Kaplan–Meier estimator was created using the demographic treatment and the rearing temperature as fixed effects, and this was plotted using the ‘ggsurv’ function, from the ‘GGally’ package (Schloerke et al., 2018) in R.

Statistical differences in reproductive output between individual‐level crosses within thermal lines and crosses between thermal lines (natives) and KSS (hybrid) individuals were analysed using GLMMs. We aimed to test for an effect of outcrossing in each of the two sets of lines, rather than for a difference between lines, so we ran two models (one for each set of thermal lines). The response variable was the number of offspring produced, with a Poisson error distribution. The combination of parents (i.e. migrant mother, migrant father or both parents native) was fitted as a categorical explanatory variable, with the within line crosses set as the baseline. We included a random effect of the ID of the thermal line of the native parent(s), and an observation‐level random effect to account for overdispersion.

To test for differences in the longevity of the offspring produced from the hybrid crosses, Cox mixed effect models were constructed, which included the treatment (male migrant, female migrant or within line cross) as a fixed effect, and the line ID of the thermal line parent as a random effect. Four models were constructed: (A) the longevity of 30°C hybrids with KSS at 38°C; (B) the longevity of 30°C hybrids with KSS at 40°C; (C) the longevity of 38°C hybrids with KSS at 38°C and (D) the longevity of 38°C hybrids with KSS at 40°C. The models were constructed with censoring, based on how many beetles were alive at the end of the experiment. For each model, a Kaplan–Meier estimator was created using the treatment as the fixed effect.

A linearised mixed model (LMM) was fitted to test for differences in the number of offspring produced by pairs of beetles from either the same, or different high temperature lines. The model was fitted with a response variable of number of offspring produced per pair over 14 days – usually, for count data, a Poisson error distribution is used, but after inspecting the residual plots and AIC values for this dataset, a Gaussian model was a better fit. The origins of the parents (same line or different lines) was fitted as an explanatory variable, with a random effect of the maternal ancestral line and the paternal ancestral line. In addition to this, differences in fitness within versus between crosses was assessed separately for each group of lines (A‐E) within the experiment using a generalised linear model (GLM) per group. For each model, the number of offspring produced per cross over 2 weeks was fitted as the response variable. The line origin of the parents was fitted as an explanatory variable with the hybrid cross in a group always fitted as the baseline.

## 3 RESULTS

### 3.1 Population‐level demographic manipulations and fitness

**FIGURE 2:**
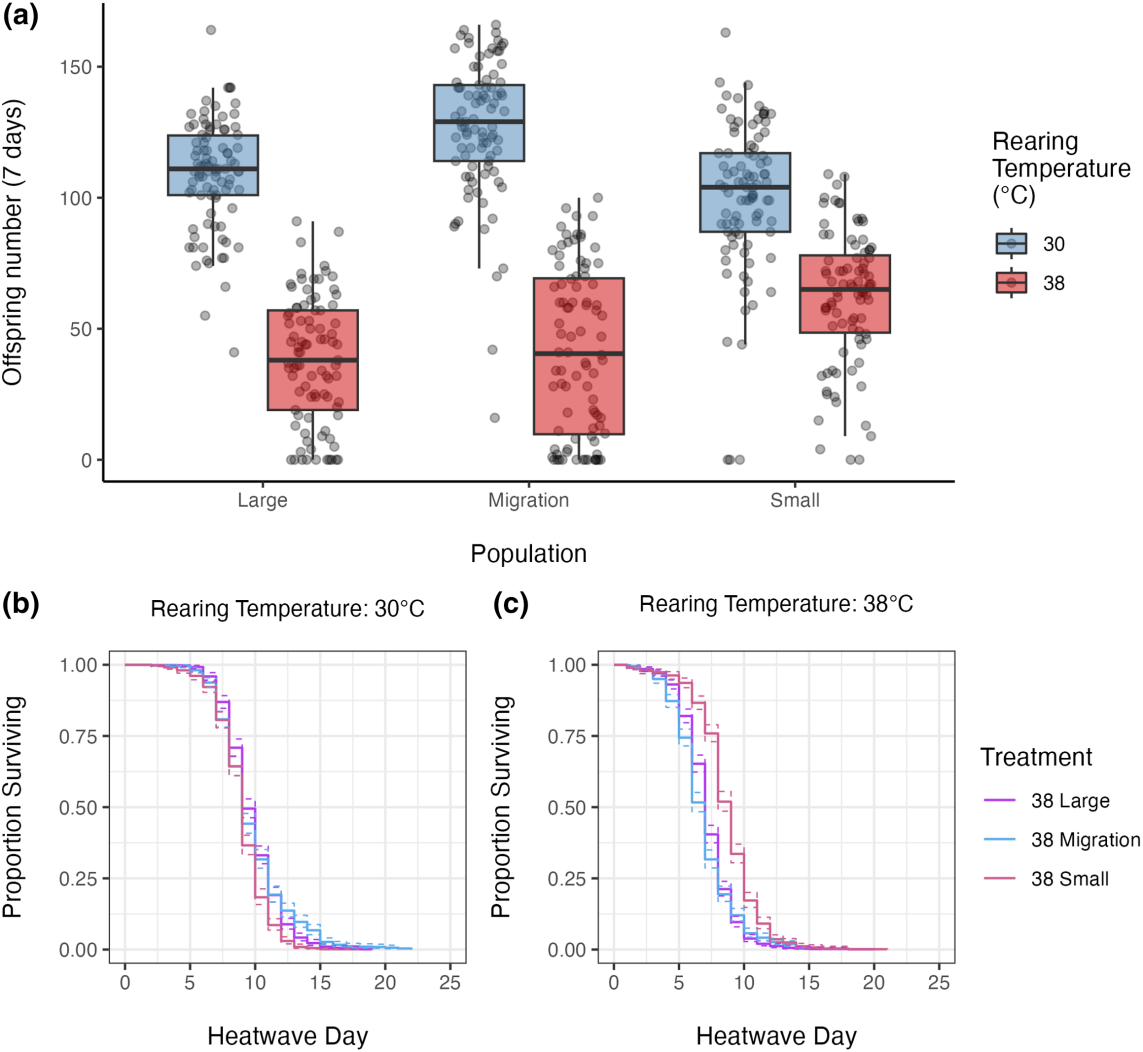
(a) The reproductive output of individual *Tribolium castaneum* females from different populations over 7 days, immediately after 24 h of mating. Each treatment consisted of 30 replicate pairs from each of the three demographic thermal lines, reared from eggs through to adulthood at either 30°C or 38°C. A rearing temperature of 38°C resulted in significantly fewer offspring produced than 30°C, although this effect was weaker in small populations. Overall, small populations produced more offspring than large. (b, c) Kaplan–Meier survival curves of *T. castaneum* from each demographic treatment through a 42°C heatwave, after 10 generations of demographic control. Beetles were reared at (b) 30°C or (c) 38°C. Dotted lines represent 95% confidence intervals. Each treatment contains three line replicates with ~300 individuals per line. Individuals reared at 38°C had reduced survival time through a heatwave. Overall, the rearing temperature had less of an effect on small populations than large or migration populations, and individuals from small populations were able to survive longest in the heatwave conditions.

Reproductive pairs produced fewer offspring when reared at the higher temperature (38°C), indicating that these conditions represent a fitness challenge (Figure 2a). Overall, there was a significant interaction between rearing temperature and demographic background (Table 1; Figure 2a). The negative effect of high temperature (38°C) on reproductive output was larger in both demographic treatments with large population size (with and without 10% migration) than in the treatment where population size remained small (Table 1; Figure 2a).

**TABLE 1:** GLMM fitted to model reproductive output of pairs of *Tribolium castaneum* from thermal regimes with different population demographics (size and migration).

Fixed effect	Estimate	SE	*Z*	Pr (>|*z*|)
Intercept	4.504	0.078	57.72	<.001
Rearing temperature (38°C)	−0.523	0.112	−4.67	<.001
Large treatment	0.161	0.110	1.46	.143
Migration treatment	0.302	0.110	2.74	.006
Large treatment × Rearing temperature	−0.835	0.159	−5.27	<.001
Migration treatment × Rearing temperature	−1.033	0.160	−6.48	<.001

*Note*: The number of offspring produced over 7 days by individual mating pairs was the response variable and there was a random effect of line (Var = 0.009) and an observation level random effect (Var = 1.778) was added to account for overdispersion. The rearing temperature and the demographic treatment were fitted as factors with control temperature (30°C) and large population size fitted as the baseline in order to separately identify the effect of population size and migration.

Survival through the 42°C heatwave condition, depended significantly upon the interaction of demographic treatment and developmental temperature (Table 2). Individuals reared at 30°C had median survival time of 9 days in all three demographic treatments (Figure 2b). When reared at 38°C, small lines had a median survival time of 9 days, whereas median survival time for both large and migration lines was 7 days. Statistically, the negative effect of higher rearing temperature (signified by higher hazard ratios at 38°C relative to 30°C) was greater in demographic treatments with large population size (with and without migration) than in the treatment with small population size (Table 2, Figure 2b,c).

**TABLE 2:** A Cox mixed effects model of survival of *Tribolium castaneum* through a 42°C heatwave, after 10 generations of small or large population sizes and developmental exposure to either 30°C or 38°C.

Fixed effect	Coefficient	Exp (coef)	SE	*Z*	*p*
Rearing temperature (38°C)	0.060	1.062	0.051	1.19	.236
Large treatment	−0.285	0.751	0.116	−2.46	.013
Migration treatment	−0.332	0.718	0.117	−2.84	.004
Large treatment × Rearing temperature	1.034	2.812	0.070	14.71	<.001
Migration treatment × Rearing temperature	1.104	3.016	0.072	15.27	<.001

*Note*: Each treatment was made up of three replicate lines (*n* ~ 300 individuals per line) and the ID of the replicate line used was fitted as a random effect (Var = 0.013). Demographic treatment and rearing temperature were fitted as factors; with large population size and control temperature (30°C) set as the baselines.

### 3.2 Outcrossing and fitness

**FIGURE 3:**
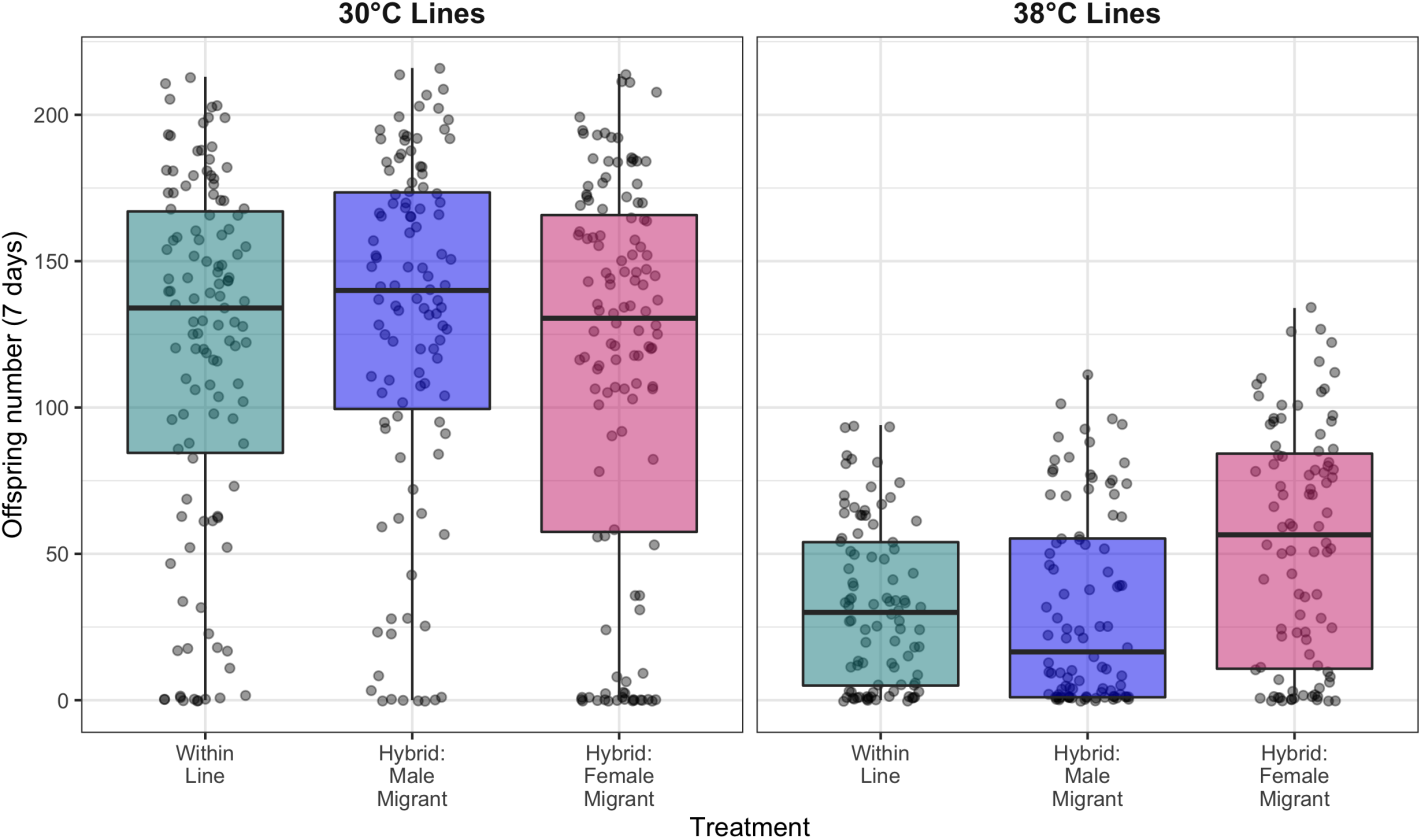
The number of offspring produced over 7 days by *Tribolium castaneum* pairs from either within one thermal line or a thermal line hybridised with a migrant from the stock population. Data are split into Control temperature lines and High temperature lines. There was no difference between the treatments in the control lines, but the high temperature lines produced significantly more offspring when crossed with a migrant female.

Overall, there was no difference in the number of offspring produced by within‐line pairs compared to pairs that included either a stock male or stock female in the control (30°C) thermal lines (Table 3, Figure 3). However, in the high‐temperature 38°C thermal lines, outcrossing of thermal line males with stock females (but not vice versa), resulted in a larger number of offspring being produced by these pairs than any other parental combination (Table 3, Figure 3). The offspring of hybrid crosses (between ‘migrant’ individuals from the KSS stocks with individuals from the thermal lines) generally had greater longevity in high temperature conditions than offspring from within‐line crosses. This effect was observed in both the 30°C control temperature thermal lines and the 38°C high temperature thermal lines, and was evident in both 38°C and 40°C heatwave conditions. However, in the 38°C condition, there were two exceptions to this, in which the longevity of hybrid offspring was no different to that of the within‐line crosses: (1) Male migrant × control female and (2) female migrant × high‐temp male. These exceptions were both for trials of longevity at 38°C, which is a less stressful temperature than the 40°C trials, and in no case was the longevity of hybrid offspring lower than the offspring from the within‐line crosses (Table 4).

**TABLE 3:** GLMMs modelling the number of offspring produced by *Tribolium castaneum* KSS stock and thermal line crosses.

Fixed effect	Estimate	SE	*Z*	Pr (>|*z*|)
**(A) Hybrids at 30°C**				
Intercept	4.425	0.165	26.79	<.001
Female migrant	−0.253	0.191	−1.32	.186
Male migrant	0.184	0.192	0.96	.339
**(B) Hybrids at 38°C**				
Intercept	2.744	0.195	14.05	<.001
Female migrant	0.538	0.245	2.20	.028
Male migrant	−0.363	0.245	−1.49	.138

*Note*: (A) ‘hybrid’ pairs between KSS migrants and 30°C thermal beetles and (B) ‘hybrid’ pairs between KSS migrants and 38°C thermal beetles. The number of offspring produced, per pair, over 7 days was the response variable. The origin of the parents (treatment) was fitted as a factor, with ‘within line crosses’ set as the baseline treatment. In both models there was a random effect of the thermal line ID used for the thermal parent beetle (VarA = 0.09, VarB = 0.08) and an observation level random effect was included to account for overdispersion (VarA = 1.85, VarB = 2.63).

**TABLE 4:** A Cox mixed effects model of *Tribolium castaneum* offspring survival of the hybrid crosses between thermal lines and KSS stock individuals (migrants).

	Coefficient	Exp (coef)	SE	*Z*	*p*
**(A) 30°C lines at 38°C**					
Male migrant	−0.201	0.818	0.051	−3.93	.057
Female migrant	−0.267	0.765	0.052	−5.12	.003
**(B) 30°C lines at 40°C**					
Male migrant	−0.264	0.768	0.050	−5.30	.001
Female migrant	−0.812	0.834	0.051	−3.56	.004
**(C) 38°C lines at 38°C**					
Male migrant	−0.069	0.934	0.034	−2.01	.045
Female migrant	−0.040	0.934	0.034	−2.01	.240
**(D) 38°C lines at 40°C**					
Male migrant	−0.129	0.879	0.055	−2.36	.018
Female migrant	−0.105	0.900	0.051	−2.07	.038

*Note*: Separate models were constructed based on the thermal line ancestral temperature and the temperature of the longevity assay. In each model, survival time (in weeks) was used as the response variable and there was a random effect of Line ID (VarA = 0.023, VarB = 0.029, VarC = 0.027, VarD = 0.016). The parent combination was fitted as a factor, with crosses within thermal lines set as the baseline.

**FIGURE 4:**
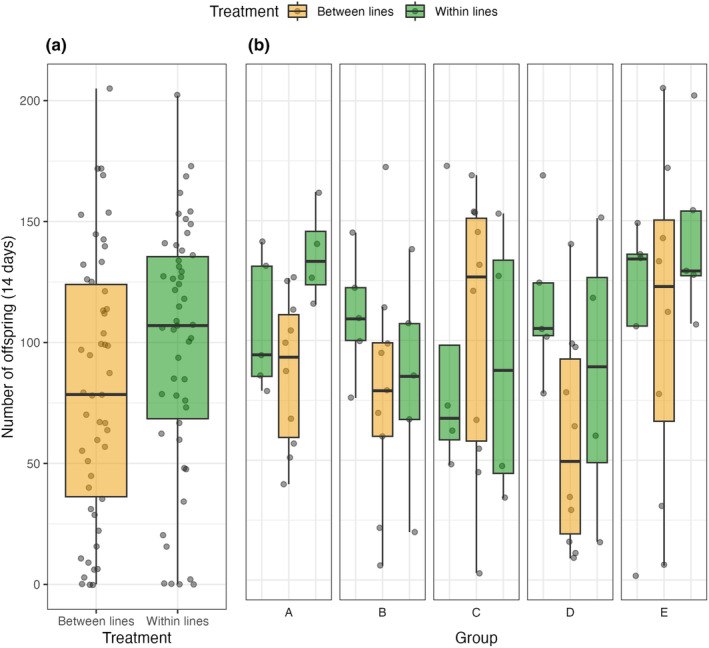
The number of offspring produced over 14 days by *Tribolium castaneum* females paired with a mate from within their own line or from another high temperature line. Panels show (a) Summary of data across all lines and combinations. (b) A breakdown of line pairings and the crosses between them. The lines were grouped and crosses between lines occurred within groups. Relative to Table 5 (below), Line 1 of each group (A–E) is plotted on the left, Line 2 on the right and their cross in the middle. Overall, there was no difference in reproductive output of within‐ or between‐line crosses.

In comparisons of reproductive fitness of within‐line and between‐line crosses for the 38°C high temperature lines, we found no overall difference in reproductive output of pairs from within one line compared with pairs composed of individuals from two different lines (Table 5; Figure 4A). Within the groups, there was also generally no difference in the reproductive output of pairs from within‐line versus between‐line crosses; the only exception was in group D where one within‐line cross showed higher reproductive output than the between line hybrid cross, with marginal significance (Table 5; Figure 4B). In this group, the difference was between the hybrid cross and only one of the parental lines.

**TABLE 5:** Five GLMs of *Tribolium castaneum* reproductive output of high temperature (38°C) thermal line pairs and hybrid crosses between them.

	Fixed effect	Estimate	SE	*t*	Pr (>|*t*|)
Group A	Intercept	86.90	12.84	6.77	.008
	Line 1	21.70	22.24	0.98	.343
	Line 2	19.10	22.24	0.86	.402
Group B	Intercept	71.70	14.87	4.82	<.001
	Line 1	15.80	27.82	0.57	.578
	Line 2	38.70	25.75	1.50	.152
Group C	Intercept	104.40	19.12	5.46	.029
	Line 1	−32.00	33.13	−0.97	.348
	Line 2	−33.20	33.13	−1.00	.330
Group D	Intercept	57.80	15.25	3.79	.002
	Line 1	11.20	26.41	0.42	.677
	Line 2	57.80	26.41	2.19	.043
Group E	Intercept	88.00	20.53	4.29	<.001
	Line 1	55.80	35.55	1.57	.135
	Line 2	17.40	35.55	0.49	.630

*Note*: Number of offspring produced over 14 days by individual mating pairs was the response variable and the line origin of the parents was fitted as a factor. The hybrid cross was always fitted as the baseline. There was no crossing between groups (e.g. Group A contained two lines and the crosses between these lines only). The replicate lines used were all maintained in the same way at 38°C for the same length of time.

## 4 DISCUSSION

We performed an in‐depth experimental investigation into the consequences of outcrossing and genetic rescue and, overall, found no evidence that increases in population size, migration or outcrossing improved fitness in *T. castaneum* populations. Despite some evidence supporting an increase in hybrid offspring longevity at the individual level, there was no indication that increases in population size and levels of migration could improve fitness. Further to this, crossing individuals between independent replicates of the high temperature lines did not result in increases in reproductive output or survival. These results were unexpected, as the thermal lines had been maintained for over 50 generations at small population sizes, so were likely to be suffering from some level of inbreeding depression. However, in these conditions, it seems that fitness was not improved, but was in fact impaired, by the attempts at rescue.

The small thermal line populations could be considered a bottlenecked version of the large stock populations (the source of the migrants for this study), where the number of adults founding each generation was reduced from 600 (in the KSS stocks) to 100. Additionally, due to strong selection pressures, especially at the start of experimental evolution, it is likely that the 38°C high temperature lines underwent more significant bottlenecks than the 30°C control lines. As such, population sizes were, for >50 generations, as small as other small populations considered in similar tests of genetic rescue (e.g. Hufbauer et al., 2015). Previous studies have shown that high temperature conditions can be stressful for *T. castaneum* and reduce their reproductive fitness (Sales, 2018; Sales et al., 2018). Therefore, it is likely that effective population size was reduced before adaptation could take place. Early in their evolution at 38°C, these thermal lines produced approximately half the number of adults compared to their 30°C equivalents, and some replicates did not produce enough adults to maintain them (Dickinson, 2018), however we lack the data on exact population sizes that would allow us to assert this definitively. As such, levels of inbreeding and genetic drift may have been higher in 38°C populations relative to controls which, in turn, could have resulted in reduced fitness or adaptive capacity (Lande & Barrowclough, 1987).

At the whole‐population level, we found unexpected evidence of decreased fitness after the populations had been free to rapidly expand to 100 times their original size, contained within a much larger, but otherwise identical, habitat. Much of the research into population expansion focuses on spatial expansion and colonisation of new territories. Individuals at the edge of the expansion tend to be less fit than those remaining in the ancestral range (Peischl et al., 2013). However, this logic applies to populations undergoing range expansion into heterogeneous habitats, and numerous factors, including spatial sorting and gene surfing, can create changes in fitness (Miller et al., 2020). The format of this study did not necessarily enable colonisation of novel areas, but allowed expansion into the same habitat but on a much larger scale. Theoretical work has shown that a rapid increase in population number can result in some interesting genetic effects, including an excess of rare mutations (Slatkin & Hudson, 1991; Zeng & Charlesworth, 2010), and a decrease in linkage disequilibrium (Slatkin, 1994). This effect has been observed in humans (Keinan & Clark, 2012), however, models predict that, although there are a larger number of deleterious variants present, the more detrimental ones are more effectively purged in a large population, so the resulting individual fitness should be similar (Gazave et al., 2013). Experimental research is required to supplement theory and explore these issues in more detail and across different conditions. An increase in population size often results in a change in density, however, we were able to control for density here by scaling up the size of the population enclosure and resources available in proportion to the number of adults founding each generation.

It is generally accepted that gene flow into a small population can increase genetic diversity and levels of fitness (Frankham, 2016), and high‐profile success stories of genetic rescue exist (Ingvarsson, 2002; Madsen et al., 1999). However, we found no evidence that 10 generations of migration into previously bottlenecked populations resulted in increased fitness. There may be several reasons why we fail to observe an increase in fitness. Firstly, migrant genotypes may not have introgressed into the recipient population – gene flow as a result of migration is not guaranteed, as migrant individuals may fail to reproduce viably (Turček & Hickey, 1951), but it is likely that at least some gene flow occurred in these lines, as genome sequences of 12 individuals from each migration and large line show marked differences in heterozygosity and evidence of introgression (R. Lewis & L. G. Spurgin, unpublished data). However, gene flow may not always result in increased fitness, as high levels of migration into a population can disrupt local adaptation, possibly resulting in offspring with reduced fitness (Storfer, 1999). In these cases, migration will oppose naturally selected optima, resulting in suboptimal fitness, a phenomenon known as ‘migration load’ (Lenormand, 2002). It is possible that the more diverse KSS migrants in this study were also less fit than the thermal lines adapted to higher temperatures, and this gene flow would therefore not confer any fitness benefits to the population. However, if this were the case, we would expect to observe some fitness cost of such crosses at the individual level (Edmands, 2007). Instead, we found that crossing males from high temperature thermal lines with migrant females resulted in increased fitness at high temperature, but there was no such effect in any of the other crosses. Furthermore, in almost all of the treatments, the offspring resulting from these crosses had increased longevity in trials at high temperatures, compared with the 30°C controls.

Migration into a population can also lead to ‘swamping’ of the local gene pool if a migrant has particularly high short‐term productivity, resulting in a breakdown of local adaptation (Hedrick & Fredrickson, 2010; Lenormand, 2002), or an introduction of deleterious alleles (Hedrick et al., 2014). This study maintained a 10% migration rate into the population for 10 generations, which is arguably unrealistically high. When undertaken for conservation, genetic rescue attempts are usually a single gene flow event at a higher frequency (e.g. Florida Panther, ~30%; Johnson et al., 2010); Mexican wolves, three lineages of three, two and two individuals merged (Hedrick et al., 1997). However, there are arguments that long‐term genetic restoration (low‐level gene flow over several generations) is the more effective approach to salvaging vulnerable populations (Adams et al., 2011). Yet, such sustained gene flow may be more likely to result in genetic swamping (Haygood et al., 2003). We found that gene flow at the population‐level did not affect fitness, despite evidence that individual outcrossing showed some limited positive effects. When investigating the individual‐level effects of outcrossing, we found that female migrants mating with native males resulted in more progeny than any other pairing. However, we found no fitness benefits when high temperature females were mated with either a high temperature male from an independent line, or a migrant male. These findings are consistent with a scenario in which initially increased fitness of migrant individuals can be lost in future generations, either as a result of the breakdown of local adaptation, or as a result of the introduction of deleterious alleles, which then spread. Evidence from other studies in *Tribolium* suggest that populations receiving migration (including comparably high levels of *m* = 0.1) still show rescue effects (Durkee et al., 2024; Hufbauer et al., 2015). However, these studies differ from the present case which is introducing migrants to a populations which has been pre‐exposed to the challenging environment for >50 generations, but show no evidence of increased fitness in that environment.

One explanation of the sex‐specific difference in fitness of outcrossed pairs may be that sex‐specific adaptation has occurred in the lines. If male‐specific function is thermally‐sensitive, and males from the 38°C high temperature lines have adapted to function at higher temperatures, the migrant (unadapted) males will introduce poorly adapted male traits for high temperature, leading to outbreeding depression in such crosses. This theory is supported by recent work on *T. castaneum* showing that male reproduction is especially sensitive to temperature (Sales et al., 2018). However, if this were the case, we would also expect to see an increase in fitness due to low levels of inbreeding when crossing between ‘adapted’ thermal lines compared to within‐line crosses. The lines had been separated for >50 generations of experimental evolution, so we expect genetic drift in these smaller populations to have caused them to differentiate enough that back‐crossing between them would have shown some genetic rescue from inbreeding. However, we found no difference in reproductive fitness between parents crossed from the same thermal line compared with parents crossed from different thermal lines (intra‐line and inter‐line crosses, respectively). An alternative explanation is that the high‐temperature females were maladapted, reducing their fecundity. This idea is supported by the lack of improvement in the thermal lines when the reproductive pairs were of mixed thermal line origin. The data also suggest that this potential female maladaptation is not an artefact of drift, in which case it would not be evident across so many lines, but instead is likely to be the result of a trade‐off or incomplete adaptation. Although there is some evidence that female reproduction can be affected by temperature (Berger et al., 2008; Irwin & Lee, 2000), there is little evidence in the literature that female fertility is more vulnerable than male fertility with regards to high temperature (Iossa, 2019; Sales et al., 2018), so further work in this area is needed to investigate the impact of high temperature on sex‐specific reproductive fitness and its physiological basis. Sex differences in gamete production are an obvious way in which temperature might affect males and females differentially – assays of reproductive fitness following heatwaves during different stages of development might shed light on whether females suffer disproportionately from developmental heatwaves due to gametic damage.

It remains a possibility that, despite being small for >50 generations, the small thermally selected populations from which we started may not have been suffering from significant inbreeding depression and their failure to increase in fitness in the challenging environment was due to some other factor. This may explain why the findings of this study diverge from those which show a strong evidence of genetic rescue (e.g. Hufbauer et al., 2015). In that scenario, lack of fitness in the challenging environment might be due to 38°C being above the limit of possible thermal adaptation, or perhaps the route to adaptation being one that our assays were unable to detect.

There is some debate amongst population geneticists with regards to the ideal population structure for conservation management (Simberloff & Abele, 1976). The argument here is whether it is better to have a single large mixing population, or several small populations between which migration can occur (Diamond, 1975; Miller‐Rushing et al., 2019). Findings of this study suggest that neither is necessarily better. Although we have found evidence that large populations were less fit than small ones, we did not find evidence that mixing small populations improves fitness, although there was no cost to this mixing. We suggest that the genetic backgrounds of small populations to be mixed may play an important role in the success of genetic rescue attempts. Populations that are genetically very similar are likely to experience little to no effect of mixing. However, if the differences between the populations are too great, outbreeding depression may occur (Lynch, 1991). These effects have been observed in both animals (e.g. the Arabian oryx, Oryx leucoryx; Marshall & Spalton, 2000) and plants (e.g. the common primrose, *Primula vulgaris*; Barmentlo et al., 2018).

In this study, we aimed to explore the role of demography (through habitat size and migration) on adaptive potential and fitness. While size is clearly an important factor for population success (Hoffmann et al., 2017), we find here that issues arising from past bottlenecks can persist, or be exacerbated, even in a large population. We find no evidence that evolutionary rescue can address this on relatively short time scales. To facilitate population rescue, assisted migration can be implemented in the hope that genetic rescue may follow, however, we also found no evidence of this effect. This study highlights the need for more experimental research into the factors underlying the success of demographic and genetic interventions undertaken for conservation purposes and for studies exploring physiological basis of thermal tolerance limits in insects.

## AUTHOR CONTRIBUTIONS

**Rebecca Lewis:** Conceptualization (equal); data curation (equal); formal analysis (lead); investigation (lead); methodology (lead); project administration (equal); visualization (equal); writing – original draft (lead); writing – review and editing (supporting). **Michael D. Pointer:** Data curation (equal); investigation (supporting); visualization (equal); writing – original draft (supporting); writing – review and editing (lead). **Lucy Friend:** Investigation (supporting); methodology (supporting). **Matthew J. G. Gage:** Conceptualization (supporting); funding acquisition (supporting); supervision (supporting). **Lewis G. Spurgin:** Conceptualization (equal); funding acquisition (lead); investigation (supporting); supervision (lead); writing – original draft (supporting); writing – review and editing (supporting).

## CONFLICT OF INTEREST STATEMENT

The authors have no relevant financial or non‐financial interests to disclose.

## Data Availability

The data to support the findings of this study are available in Dryad https://datadryad.org/stash/share/oF9PqTY8SIdNse‐UDcpcG‐AyG9_xmJJ68nqK6ZU4WEg.
